# Lumbosacral Transitional Vertebra among Patients Visiting the Department of Orthopaedics in a Tertiary Care Centre: A Descriptive Cross-sectional Study

**DOI:** 10.31729/jnma.8011

**Published:** 2023-02-28

**Authors:** Suman Kumar Basel, Ram Krishna Barakoti, Rajesh Kumar Chaudhary, Babu Kaji Shrestha, Deepak Kaucha, Sanjib Rijal

**Affiliations:** 1 Department of Orthopaedic Surgery, B&B Hospital, Gwarko, Lalitpur, Nepal; 2 Department of Orthopaedic Surgery, Chitwan Medical College and Teaching Hospital, Bharatpur, Chitwan, Nepal

**Keywords:** *lumbar vertebrae*, *orthopedics*, *prevalence*

## Abstract

**Introduction:**

Lumbosacral transitional vertebra is a normal anatomical variant at the L5-S1 junction with an incidence as high as 4-36%. This alteration results in incorrect identification of vertebral segments leading to wrong surgery. The aim of the study was to find out the prevalence of lumbosacral transitional vertebra among patients visiting the department of orthopaedics in a tertiary care centre.

**Methods:**

A descriptive cross-sectional study was conducted from 11 September 2021 to 31 May 2022, after receiving ethical clearance from the Institutional Review Committee (Reference number: IRC-2021-9-10-09). The patients with plain radiographs of the lumbosacral spine (anteroposterior view) were assessed and evaluated by a fellow and consultant of the orthopaedic spine and classified as per Castellvi's radiographic classification. Convenience sampling was done. Point estimate and 95% Confidence Interval were calculated.

**Results:**

Among 1002 patients, lumbosacral transitional vertebra was detected in 95 (9.48%) patients (9.40-9.56, 95% Confidence Interval). Out of 95 (9.48%) patients with the lumbosacral transitional vertebra, 67 (70.53%) had sacralisation and 28 (29.47%) had lumbarization. The mean age of the patients at the time of the study included in the study was 41.6±15.12 years (range 18-85 years). The lumbosacral transitional vertebra was more common in females than males. According to the Castellvi classification, type IIa was the most common type 47 (49.47%).

**Conclusions:**

The prevalence of lumbosacral transitional vertebra was similar to other studies done in similar settings.

## INTRODUCTION

Lumbosacral transitional vertebra (LSTV) are normal anatomical variants at the L5-S1 junction. The reported incidence in the general population is as high as 4-36%.^1^ It includes lumbarization of the most superior sacral segment and sacralization of the lowest lumbar segment.^2^ Today, LSTV can be identified using various imaging modalities like plain radiographs, Computed Tomography (CT), and Magnetic Resonance Imaging (MRI).^3,4^ However plain radiographs are a simple cost-effective investigation done routinely. LSTV can be identified on anteroposterior lumbosacral spine /Ferguson view and kidney ureter bladder (KUB) radiographs.

LSTV is usually associated with low back pain termed "Bertolloti syndrome". Presence of LSTV result in incorrect identification of vertebral segment which may lead to poor clinical correlation and wrong-level surgery.^5^ Knowledge of local prevalence rates can help physicians/surgeons avoid unforeseen complications.

The aim of the study was to find out the prevalence of lumbosacral transitional vertebra among patients visiting the department of orthopaedics in a tertiary care centre.

## METHODS

This descriptive cross-sectional study was conducted from 11 September 2021 to 31 May 2022, after receiving ethical approval from the Institutional Review Committee (Reference number: IRC-2021-9-10-09) of B&B Hospital, Lalitpur, Nepal. X-rays of all patients who underwent plain radiographs of the lumbosacral spine (anteroposterior view) were assessed after patient consent.

The sample size was calculated using the formula:


$$\begin{array}{ll} \text{n} & ={\text{Z}}^{2}\times \frac{\text{p}\times \text{q}}{{\text{e}}^{\text{2}}}\\ & =1.96^{2}\times \frac{0.147\times 0.853}{0.05^{2}}\\ & =772 \end{array}$$

Where,

n = minimum required sample size
Z = 1.96 at 95% Confidence Interval (CI)
p = prevalence of lumbosacral transitional vertebra taken from the previous study, 14.7%^1^
q = 1-p
e = margin of error, 5%

The minimum sample size calculated was 772. However, 1002 patients were included in the study.

Plain radiographs lumbosacral spine of 1028 patients were evaluated. 1002 patients met the inclusion criteria, and 26 patients were excluded. Radiographs of patients with good-quality lumbosacral spine were included in the study. A radiograph with clear visibility of the last rib's vertebral body articulation, all lumbar transverse process, and the complete sacral wing was classified as good quality X-ray. And radiographs with no to poor visibility of above mention structures, previous lumbosacral surgery, and destruction of vertebra due to any reason i.e., trauma, or tumour were classified as poor X-ray.

A structured tool was used to record patient age, sex, and X-ray findings. Findings of lumbosacral vertebrae include the number of lumbar vertebrae, craniocaudal measurement of the transverse process of L5 vertebra, pseudo arthrosis and/or bony fusion of L5 transverse process with the sacrum (unilateral or bilateral). The twelfth thoracic vertebra (T12) was identified as the vertebra to which the lowest rib is attached. Then lumbar vertebrae were subsequently numbered craniocaudally. X-rays were evaluated by an Orthopaedic spine fellow and Orthopaedic spine consultant. LSTV was determined as per Castellvi radiographic classification and recorded.^2^

Data were entered and analysis was done in Microsoft Excel 2013. Point estimate and 95% CI were calculated.

## RESULTS

Among 1002 patients, LSTV was detected in 95 (9.48%) (9.40-9.56, 95% CI) patients. Among the patients with LSTV, 67 (70.52%) had sacralisation and 28 (29.47%) had lumbarisation. The mean age of the patients at the time of the study was 41.6±15.12 years (range 18-85 years).

According to the Castellvi classification, 47 (49.5%) patient was categorized under type IIa of Castellvi classification accounting for almost half of all patients (Table 1).

**Table 1:** Distribution of patients as per Castellvi classification (n= 95).

Castellvi classification	Number of patients n (%)
I a	7 (7.37)
I b	1 (1.05)
II a	47 (49.47)
II b	14 (14.74)
III a	13 (13.68)
III b	8 (8.42)
IV	5 (5.26)

**Figure 1:**
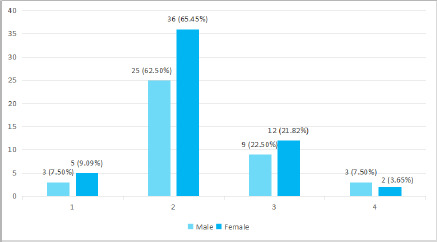
Sex-wise Castellvi classification (n= 95).

LSTV was more common in females than males. Among 95 patients, 55 (57.89%) were female and 40 (42.11%) males (Figure 1).

## DISCUSSION

The prevalence rate reported in our study was 9.48%, which was lower compared to similar studies conducted in Nepal.^1,6^ However, in an MRI-based study,^7^ the LSTV prevalence was 3.8%, much lower as compared to our study. Similarly, other similar studies from India and Saudi Arabia had lower prevalence as compared to this study. A study in Australia reported a similar prevalence as compared to our study.^8^ The prevalence of LSTV has a wide prevalence rate ranging from 4-36% since it was first reported in 1977.^1^ This wide variability can be due to interobserver variability, the imaging technique used, and the population being studied.

Sacralization was more common than lumbarization, similar to other studies.^1,6,9^ A study conducted in Australia reported lumbarization to be more common than sacralization, in contrast to our study, which could be because of the exclusion of type I LSTV in his study causing decreased recorded cases of sacralization.^8^

LSTV was more common in females in our study which was similar to other studies conducted in a similar setting in Nepal.^1,6^ As per other studies carried out in India,^10^ USA,^11^ Saudi,^12^ and Turkey^13^ reported that males had a higher prevalence rate.

Type II LSTV was the most common type in this study like the study conducted in India^10^ in a similar setting. In contrast to our study, Type I was the most common type in a study conducted in Nepal.^1,6^

A study conducted in USA^11^ showed similar findings with additional findings of Type IV with high prevalence as Type II and severity of low back pain. Our study had a high number of Type II patients because this study analysed lumbosacral spine radiographs only and most patients who undergo these X-rays have back pain. The association between LSTV and back pain was first described in 1917 by Bertolloti, although the association still remains controversial.^14,15^ The actual clinical symptom and indications for x-rays were unknown, hence the relation between back pain and LSTV could not be explored. This can be done in further studies.

This is a single hospital-based study, so the prevalence rate cannot be generalized to the community. Also, this study included patients visiting the hospital and getting lumbosacral spine X-rays (assumed mostly for back pain), which may not truly reflect the general population. Whole spine X-rays of the patients were not analyzed so an extra lumbar rib could have been missed leading to an error.

## CONCLUSIONS

The prevalence of lumbosacral transitional vertebra was similar to other studies done in similar settings. LSTV is a common anatomical variation occurring at the lumbosacral junction. Hence, all surgeons and treating physicians should be observant, to identify the anatomical variant.
